# Gα modulates salt-induced cellular senescence and cell division in rice and maize

**DOI:** 10.1093/jxb/eru372

**Published:** 2014-09-16

**Authors:** Daisuke Urano, Alejandro Colaneri, Alan M. Jones

**Affiliations:** ^1^Department of Biology at the University of North Carolina, Chapel Hill, NC, 27599-3280, USA; ^2^Department of Pharmacology at the University of North Carolina, Chapel Hill, NC, 27599-3280, USA

**Keywords:** Abiotic stress, maize, NaCl stress, plant heterotrimeric G protein, rice, sodium perception.

## Abstract

The plant G-protein network, comprising Gα, Gβ, and Gγ core subunits, regulates development, senses sugar, and mediates biotic and abiotic stress responses. Here, we report G-protein signalling in the salt stress response using two crop models, rice and maize. Loss-of-function mutations in the corresponding genes encoding the Gα subunit attenuate growth inhibition and cellular senescence caused by sodium chloride (NaCl). Gα null mutations conferred reduced leaf senescence, chlorophyll degradation, and cytoplasm electrolyte leakage under NaCl stress. Sodium accumulated in both wild-type and Gα-mutant shoots to the same levels, suggesting that Gα signalling controls cell death in leaves rather than sodium exclusion in roots. Growth inhibition is probably initiated by osmotic change around root cells, because KCl and MgSO_4_ also suppressed seedling growth equally as well as NaCl. NaCl lowered rates of cell division and elongation in the wild-type leaf sheath to the level of the Gα-null mutants; however there was no NaCl-induced decrease in cell division in the Gα mutant, implying that the osmotic phase of salt stress suppresses cell proliferation through the inhibition of Gα-coupled signalling. These results reveal two distinct functions of Gα in NaCl stress in these grasses: attenuation of leaf senescence caused by sodium toxicity in leaves, and cell cycle regulation by osmotic/ionic stress.

## Highlight text

This work reveals two distinct functions of Gα in NaCl stress in rice and maize: attenuation of leaf senescence caused by sodium toxicity in leaves, and cell cycle regulation by osmotic/ionic stress.

## Introduction

Sodium chloride (NaCl), the most abundant salt in nature, damages plants through ion toxicity and high osmolality, resulting in a decline in agricultural productivity. The osmotic phase rapidly inhibits growth of young immature leaves by decreasing cell proliferation and delaying cell elongation, while the ionic phase gradually induces cellular senescence of mature leaves (Munns and Tester, 2008; Taleisnik *et al.*, 2009; Geng *et al.*, 2013; Roy *et al.*, 2014). Ionic toxicity is primarily due to the sodium ion rather than the chloride ion, because sodium has a lower threshold for toxicity than chloride. Plants cope with high salinity by three mechanisms: tolerance to osmotic stress; exclusion of sodium from roots or secretion of from leaves; and tolerance to accumulated sodium in shoots (Munns and Tester, 2008). Sodium exclusion occurs by sodium-hydrogen anti-transporters on the plasma membrane (Ji *et al.*, 2013). However molecular mechanisms conferring osmotic and toxic tolerance in shoots, specifically signal mediators between salinity and development, are poorly understood (Roy *et al.*, 2014).

The heterotrimeric G protein, composed of Gα, Gβ, and Gγ subunits, serves as a physical coupler between membrane receptors and intracellular signalling proteins (Urano *et al.*, 2013). Upon nucleotide exchange of GDP for GTP on the Gα subunit, the heterotrimer dissociates into two active signalling units: a GTP-bound Gα and a Gβγ dimer. The active Gα returns to the basal state by hydrolysing GTP to GDP. *Arabidopsis* Regulator of G Signalling 1 (AtRGS1) is a seven transmembrane (7TM) protein, a cytoplasmic Regulator of G Signalling (RGS) domain that accelerates GTP hydrolysis by Gα, therefore suppressing G-protein activity (Chen *et al.*, 2003; Johnston *et al.*, 2007). The G-protein network regulates development, hormone responses, stomatal opening and closure, innate immunity, sugar sensing, and several abiotic stress responses in *Arabidopsi*s (Urano *et al.*, 2013). *Arabidopsis* loss-of-function mutants of Gα or RGS1 attenuate NaCl-induced senescence, while the Gβ-null mutants are hypersensitive to NaCl (Colaneri *et al.*, 2014). The mechanism of G-protein mediation of NaCl tolerance is unclear; however, NaCl indirectly induces RGS1 endocytosis (Colaneri *et al.*, 2014) thus allowing self-activation of Gα on the plasma membrane (Urano *et al.*, 2012b; Fu *et al.*, 2014).

A study of the G-protein-dependent mechanism of salt tolerance in rice and maize is important not only because these are major crops but because the 7TM-RGS gene does not exist in rice and maize (Urano *et al.*, 2012a) and therefore these species offer an opportunity to discover a novel G-protein activation mechanism. The loss of an RGS1-mediated pathway in rice and maize may have reshuffled the G-protein networks and evolved the physiological functions of G proteins. It is currently unknown (1) if a G-protein pathway architecture lacking a 7TM-RGS regulator mediates salt stress responses, (2) if G proteins control cell division and elongation suppressed by NaCl, and (3) how the G-protein network attenuates NaCl-induced senescence.

We propose that sodium accumulation in shoots induces cellular senescence through Gα activation, and that osmotic stress by high salinity inhibits cell proliferation by inactivating Gα signalling. Loss-of-function mutations of Gα attenuated leaf senescence caused by sodium toxicity without altering sodium uptake into shoots. In addition, Gα-null mutations attenuated growth inhibition caused by the osmotic component of salt stress. NaCl inhibited cellular elongation similarly in wild-type and Gα-null plants, but reduced cell division only in the wild type, probably because cell division was already suppressed to the basal level in Gα-null plants even under non-stress conditions. These results suggest that Gα functions at two different stages of plant responses to NaCl: cell cycle regulation by osmotic pressure and cell senescence by sodium toxicity.

## Materials and methods

### Preparation of salt-treated samples

**Fig. 1.**
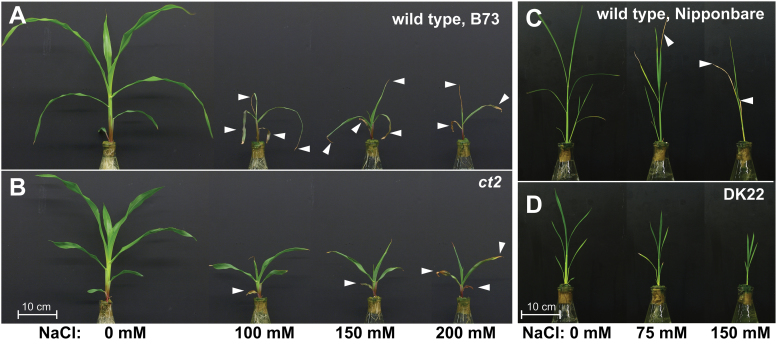
Representative maize and rice seedlings after NaCl treatment. (A) Wild-type maize, B73, (B) Gα-null maize *ct2*, (C) wild-type rice Nipponbare, and (D) Gα-null rice, DK22 seedlings were treated with the indicated concentration of NaCl in 1/4X MS liquid media for 10 d. Dead leaf tips are indicated by white arrowheads.

Rice (*Oryza sativa*) seeds of wild-type Nipponbare and the Gα-null mutant (DK22) (Fujisawa *et al.*, 1999) were sterilized and germinated under darkness at 30°C for three days. Seedlings were transferred to tap water with 0.025% MES (pH 5.7) for hydroponic growth at 28°C under a 24-h light cycle of 210–220 µmol m^–2^ s^–1^ fluence for 16h. Ten- or 11-day-old seedlings with three leaves were transferred to 1/4X Murashige and Skoog (MS) liquid media with 0.025% MES (pH 5.7) and different concentrations of NaCl (0, 75, or 150mM). The pH was adjusted with KOH. The seedlings were floated on the indicated solutions held on a styrofoam raft with A-OK Starter Plugs (Grodan®, Roemond, The Netherlands), then moved to the neck of an Erlenmeyer flask to capture images as shown in Fig. 1. After a 10-d treatment with NaCl, the fresh weights (FWs) of shoots and length of the fourth leaf sheath or blade were measured. Leaf disks were collected for analysing ion leakage and chlorophyll contents as described below. The remaining shoot was used to determine sodium and potassium contents. Maize (*Zea mays*) seeds of wild type B73 or the Gα-null mutant *ct2-ref* (*ct2*) (Bommert *et al.*, 2013) were germinated and grown in soil for 2–3 days. Prior to the stage of first-leaf unfurling, seedlings were transferred to 0.025% MES hydroponic solution (pH 5.7). Seedlings at the one-leaf stage were transferred to 1/4X MS liquid media containing 0.025% MES (pH 5.7) and 0, 100, 150, or 200mM NaCl. After 10 d of treatment, the FWs of shoots and length of the third leaf sheath or blade were measured. The growth conditions of temperature and light were the same as those for rice seedlings.

### Cell elongation and cell number in leaf sheath

Negative impressions of the maize leaf surface from areas located ~5–20mm below the third leaf collar were obtained using a dental impression material, Aquasil® Ultra LV Type 2 (DENTSPLY Caulk, Milford, DE, USA). Positive impressions were created by coating the rubber dental mould with clear liquid nail polish, which was then peeled off and mounted on a glass slide. Topography of the surfaces was observed with an Olympus OX81 microscope using a 10× objective lens. Length of pavement cells in the intercostal zone was measured using Image J software. Reported cellular lengths represent the average of >130 cells from three independent plants. The number of cells forming the third leaf sheath was estimated by dividing the length of leaf sheath by the average length of epidermal cells. Samples that required drying were kept in a fume hood until dry.

### Chlorophyll content measurement

Two rice leaf disks of 5-mm diameter were obtained from the widest region of the second and third leaves. Leaf disks were homogenized in 1.0ml of 80% acetone using a bench-top homogenizer (MP Bioscience FastPrep-24), then incubated for more than 24h at room temperature. The extracts were centrifuged for 5min at 15 000 *g*. Absorbance of the supernatant was measured at wavelengths 645, 646, and 663nm (A_645_, A_646_, and A_663_) with a Shimadzu^TM^ UV-3000 dual-wavelength double-beam spectrophotometer. Complete scans were obtained to assure that the predominant absorbance was from chlorophyll. Samples having absorbance greater than 1.5 were diluted by half with 80% acetone and reevaluated. Chlorophyll concentration was estimated following Lichtenthaler’s equations shown below (Lichtenthaler and Wellburn, 1983) and Arnon’s equation (see Supplementary material; Arnon, 1949):

$$\text{Chlorophyll a }\left[{\text{mg l}}^{–\text{1}}\right]\;=\text{ 12}.\text{21 }\times {\text{ A}}_{\text{663}}–\text{ 2}.\text{81 }\times {\text{ A}}_{\text{646}}$$

$$\text{Chlorophyll b }\left[{\text{mg l}}^{–\text{1}}\right]\;=\text{ 2}0.\text{13 }\times {\text{ A}}_{\text{646}}–\text{ 5}.0\text{3 }\times {\text{ A}}_{\text{663}}$$

$$\text{Total chlorophyll }\left[{\text{mg l}}^{–\text{1}}\right]\;=\text{ Chlorophyll a }+\text{ Chlorophyll b}$$

Chlorophyll content was expressed as µg of total chlorophyll per two leaf disks. Reported values represent the average of twelve or more samples.

### Ion leakage measurement

Leaf disks of 6.2-mm diameter were collected near the widest part of the second and third leaves of maize seedlings. Each disk was immersed in 1ml of distilled water in a 48-well plate. The plate was incubated for 15–20h with shaking gently under darkness, then a 870 µl aliquot was removed from each well and transferred into empty wells. The conductivity in µS cm^–1^ was measured with a conductivity meter, VWR E C Meter 1054. Averages of eight or more samples were used for statistical analyses.

### Ion content measurement

Rice and maize shoots were dried in an oven at 80°C for 48h, weighed, and pulverized using a mortar and a pestle. Approximately 1.3g of maize samples were digested in 2ml of 70%-saturated nitric acid in 15-ml conical tubes. For rice, the whole sample was digested with 0.5ml nitric acid solution. After 24h incubation at 25°C followed by 4h at 55°C, the tubes were centrifuged for 10min at 1600 *g*, and 143 µl of supernatants was diluted by 5ml of 18 megohm water. Samples were filtered through a Millipore φ0.22 µm polyethersulfone filter. Concentration of sodium and potassium were determined by inductively-coupled plasma (ICP) mass spectrometry, using a Varian^TM^ 820-MS instrument. Samples containing ions greater than 2 ppm were diluted with 2% nitric acid to place the concentration within the linear sensitivity range of the instrument and then re-evaluated. Ion content is presented as mg ions g^–1^ dry tissue (DW).

### Statistical analyses

Quantitated data were analysed by one-way analysis of variance (ANOVA) with subsequent analyses by the Tukey’s multiple comparison. Significant differences between wild-type and Gα-null plants under the same NaCl treatment are shown with symbols of n.s. (*P* ≥ 0.05), † (*P* < 0.05) or †† (*P* < 0.01). Significant differences of NaCl groups to non-NaCl control groups are shown with n.s. (*P* ≥ 0.05), * (*P* < 0.05) or ** (*P* < 0.01). All raw data described in the main text are provided in the supplementary material.

## Results

### Genetic ablation of rice and maize Gα genes confer salt tolerance

**Fig. 2.**
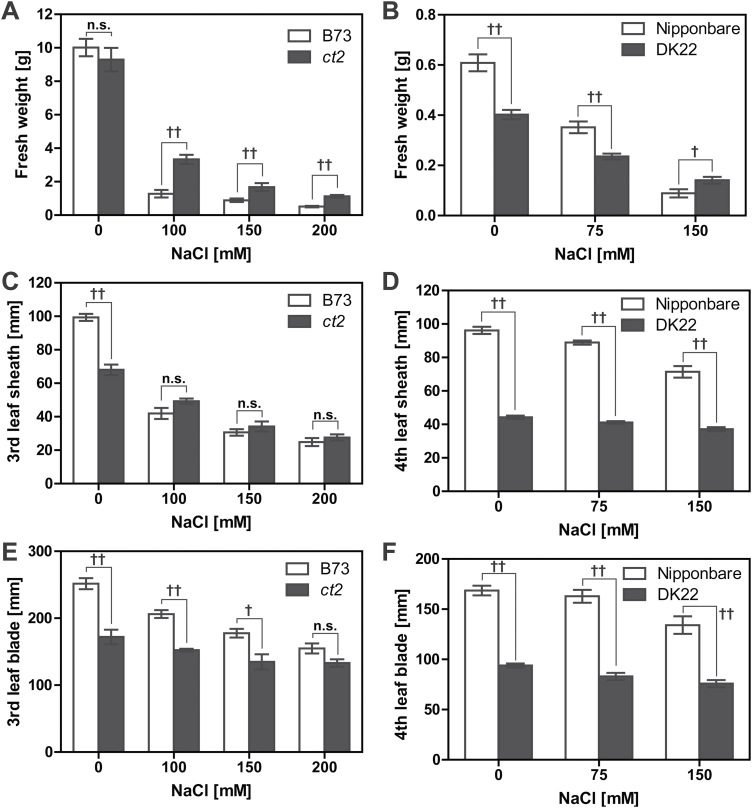
FW and leaf length of maize and rice seedlings grown with NaCl. (A, B) FW of maize or rice shoots after 10 d treatment with or without NaCl. (C–F) Length of the leaf sheath and blade of maize or rice seedlings grown with or without NaCl for 10 d. The third leaf of maize and the fourth leaf of rice were analysed. Error bars represent SEM. † or †† represents a significant difference between wild-type and Gα-null groups at a *P* value <0.05 or 0.01, respectively (one-way ANOVA with the Tukey’s multiple comparison test). n.s. means no significant difference at a *P* value of 0.05. Raw data are provided in Supplementary Figure S2.

Fig. 1 shows the seedlings on the 10th day of NaCl treatment. NaCl reduced the shoot size of maize in both wild-type B73 and the Gα-null line *COMPACT PLANT2 ct2* (Fig. 1A, B) but the *ct2* mutants were less sensitive to NaCl. B73 seedlings treated with NaCl had numerous withered leaves as indicated by arrow heads in Fig. 1 and *ct2* seedlings had fewer dead leaves. Similarly, 75 and 150mM NaCl inhibited shoot growth of both wild-type Nipponbare rice and the Gα-knockout line DK22 (Fig. 1C,D), but DK22 mutants were less sensitive to NaCl. Moreover, NaCl caused senescence only of the Nipponbare leaves. Fig. 2 shows the quantification of the qualitative results in Fig. 1. The B73 and *ct2* seedlings had comparable FWs when grown without NaCl (B73, 10.0g; *ct2*, 9.3g); however, the FWs of *ct2* seedlings were significantly greater than those of the B73 seedlings under NaCl stress (Fig. 2A). Similarly, the weights of Gα-null DK22 rice seedlings were greater than Nipponbare under the highest NaCl concentration of 150mM (Fig. 2B). NaCl shortened the leaf sheath and blade of maize (Fig. 2C, E) and rice (Fig. 2D, F). The third leaf sheath of *ct2* was shorter by 32% of the parental line B73 under control conditions (B73, 99.3mm; *ct2*, 68.0mm), but decreased less under salt stress conditions (Fig. 2C). The third leaf blade of *ct2* was also shorter by 32% for B73 under control conditions (B73, 251.6mm; ct2, 172.0mm) as shown in Fig. 2E. The blade lengths decreased to a final value of 154.9mm for B73 but only to 133.1mm for *ct2* at 200mM NaCl. The Gα-null rice DK22 also had a shorter leaf sheath (Nipponbare, 96.2mm; DK22, 44.3mm) and blade (Nipponbare, 168.6mm; DK22, 93.4mm) without NaCl treatment (Fig. 2D,F). NaCl (150mM) shortened the fourth leaf sheath of Nipponbare by 26% (71.4mm) and of DK22 by 16% (37.2mm). The fourth leaf blade of Nipponbare and DK22 were similarly shortened with 150mM NaCl (20% reduction in Nipponbare and 19% in DK22). Supplementary Figure S1 shows the effect of NaCl on maize root development. Wild-type B73 and Gα-null *ct2* maize showed comparable root length under control conditions (B73, 177mm; ct2, 182mm). NaCl shortened maize roots, but no difference was observed between B73 and Gα groups. The minimal effect of Gα may be explained by its expression profile, as rice Gα is expressed highly in shoots but much less in roots (Izawa *et al.*, 2010). These visible traits indicate that ablation of the Gα gene confers immediate NaCl tolerance to rice and maize in shoots.

### NaCl decreases cell proliferation to the basal level of the Gα mutant

**Fig. 3.**
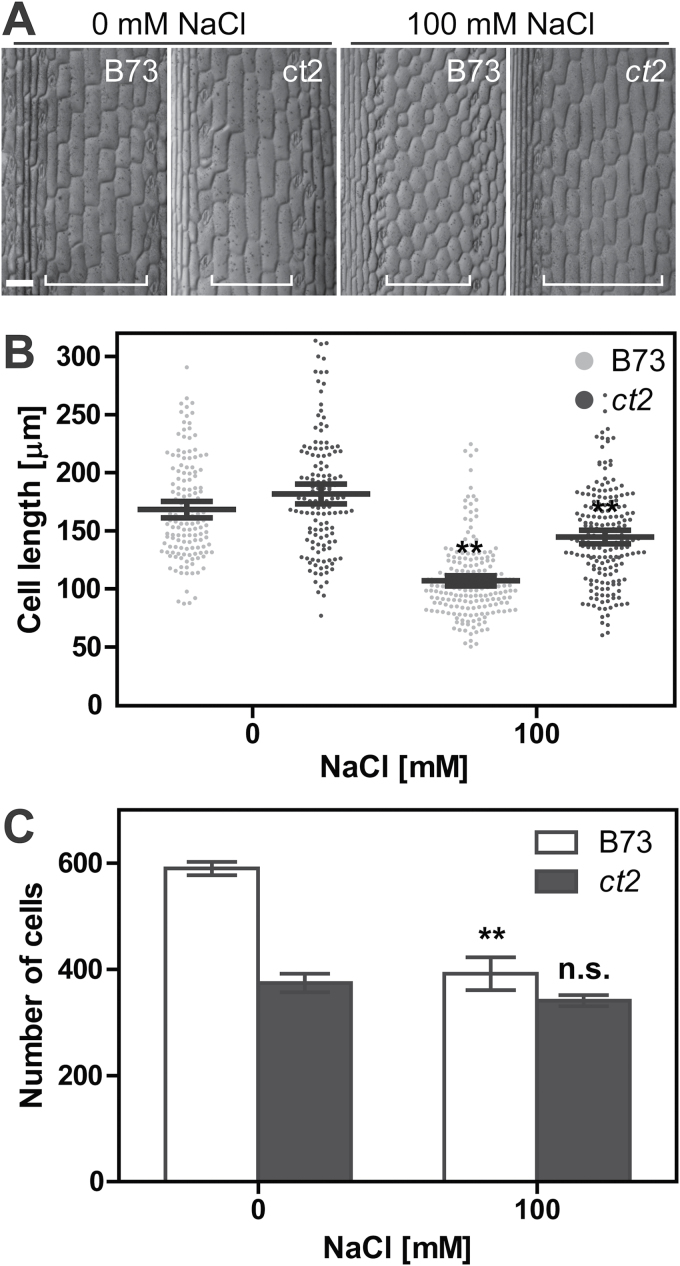
Cell expansion and division in the maize sheath. (A) Maize epidermis of the third leaf sheath grown with or without 100mM NaCl. Regions around 10mm below the leaf sheath boundary were used for imaging. Representative images are shown with scale bars of 100 µm. Pavement cells quantified in B are shown with white brackets. Note that B73 showed a severe cellular shrinkage under 100mM NaCl, probably due to proteolysis. (B) Length of leaf sheath epidermal cells after 10 d treatment with NaCl. Data are averaged over 130 cells from four or more images. Error bar represents 95% confidence interval. (C) Number of cells forming the third leaf sheath along the longitudinal axis. The number was estimated by dividing each sheath length by the averaged cellular length. Columns represent mean values with SEM. ** in panels B and C shows that the 100mM NaCl group is significantly different from the control group at *P* < 0.01 (one-way ANOVA with the Tukey’s multiple comparison test). n.s. means no significant difference (*P* ≥ 0.05). Raw data are provided in Supplementary Figure S3.

As described above (Figs. 1 and 2), while NaCl shortened both wild-type and Gα-null seedlings of maize, loss of the Gα subunit attenuated this growth inhibition. Growth is due to a combination of both cell division and elongation (Ortega *et al.*, 2006), therefore we tested if the visible phenotype of Gα is related to cell division, elongation, or both. We first examined cell elongation by imaging epidermal cells on the third leaf sheath of maize (Fig. 3A) with quantification of their cellular lengths (Fig. 3B). The *ct2* epidermal cells are 8% longer than B73 without NaCl (B73, 168.4 µm; *ct2*, 181.7 µm). NaCl (100mM) shortened B73 cells by 36% (107.0 µm), while *ct2* cells were shortened by 20% (144.6 µm). The final size of a plant organ is a function of both cell number and cell size, and the observed decrease in cell size was less than the observed decrease in organ size, suggesting that cell division was more severely suppressed than cell elongation. To quantitate average cell division activity, we determined the number of epidermal cells along the leaf sheath. The cell number was calculated by dividing sheath lengths by the averaged cellular lengths (Fig. 3C). *ct2* plants had 37% fewer epidermal cells than those in B73 (B73, 590 cells; *ct2*, 374 cells). NaCl (100mM) suppressed cell division in B73 but not in the *ct2* mutant (B73, 392 cells; *ct2*, 341 cells), probably because the cell cycling activity is already at the basal level in the *ct2* mutant. These results prompt the hypothesis that Gα signalling promotes cell cycling above a basal level and NaCl inhibits cell cycling by inhibiting Gα signalling.

### Loss of Gα attenuates cellular senescence under salt stress

**Fig. 4.**
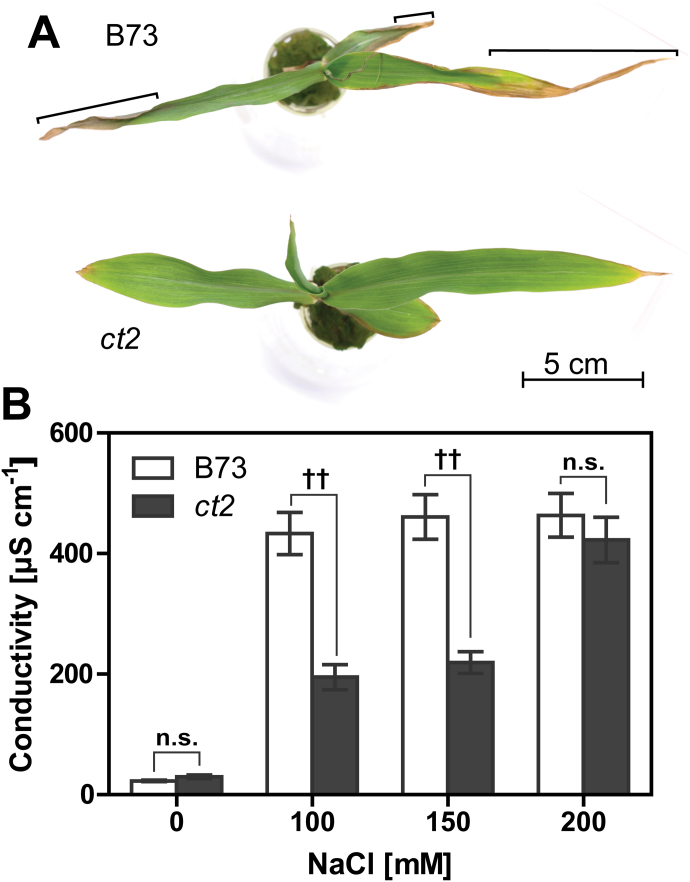
Cellular senescence of the maize leaf. Representative leaves are shown of wild-type B73 or Gα-null *ct2* leaves grown with 150mM NaCl for 7 d. Brackets indicate a dead leaf tip of B73. (B) Electrolyte leakage from the wild-type or Gα-null leaf disks were measured by electric conductivity. Data are values averaged over eight leaf measurements with SEM. † or †† represents a significant difference between wild-type and Gα-null groups at a *P* value <0.05 or 0.01 (one-way ANOVA with the Tukey’s multiple comparison test). n.s. means no significant difference (*P* ≥ 0.05). Raw data are provided in Supplementary Figure S4.

**Fig. 5.**
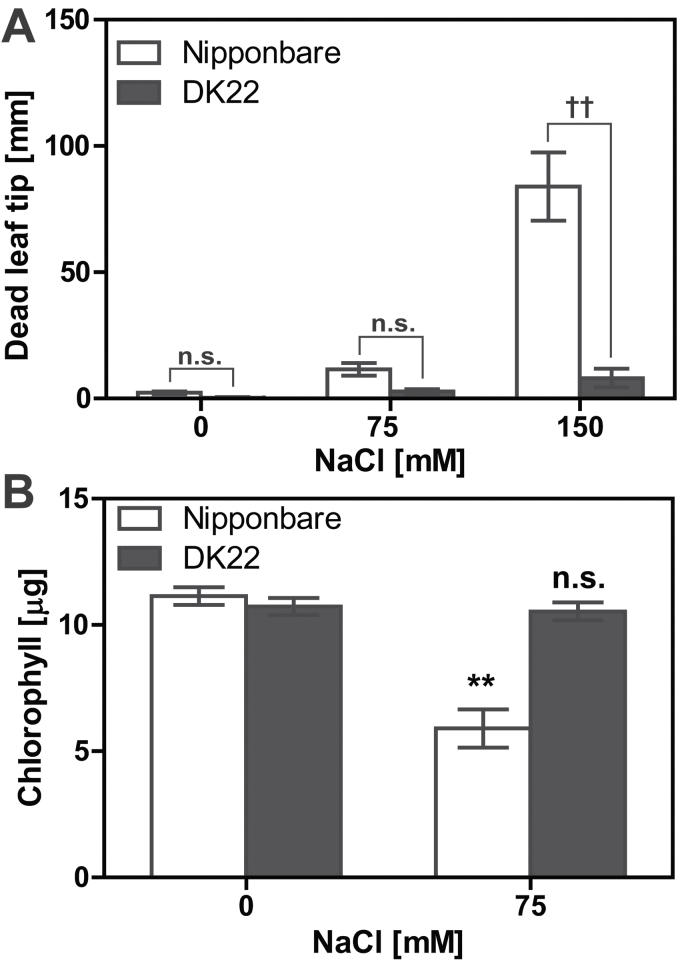
Rice leaves withered by NaCl treatment. (A) Length of withered leaf tips of wild-type or Gα-null rice grown with NaCl for 10 d. The second leaf blades were analysed. Values that averaged 13 or more measurements are shown with SEM. †† represents a significant difference between wild-type and Gα-null groups at *P* < 0.01 (one-way ANOVA with the Tukey’s multiple comparison test). (B) Total amount of chlorophyll per two leaf disks (5mm-diameter). Values are averages of 12 or more leaf disk samples and shown with SEM. ** in the B panel represents a significant difference between 0mM and 75mM NaCl groups at *P* < 0.01. n.s. represents no significant difference (*P* ≥ 0.05). Raw data are provided in Supplementary Figure S4.

Loss-of-function mutation of the Gα gene inhibited senescence (Fig. 1). Accumulation of sodium in mature leaves induces cellular senescence, manifested by dead tissue at the tips of the oldest leaves (Munns and Tester, 2008). Therefore, we quantitated the reduction of chlorophyll content (Garcia *et al.*, 1997; Chattopadhayay *et al.*, 2002) and the increase of cytoplasm ion leakage (Zhang *et al.*, 2006) as a proxy for cellular senescence. Fig. 4A shows representative seedlings of B73 and *ct2* grown with 150mM NaCl. The *ct2* mutation reduced salt-induced leaf senescence (Fig. 4A) and electrolyte leakage (Fig. 4B). While little electrolyte leakage was detected from B73 or *ct2* leaf disks grown without added NaCl (B73, 23.0 µS cm^–1^; *ct2*, 29.8 µS cm^–1^), the conductivity of B73 samples increased to 433.1 µS cm^–1^ with 100mM and conductivity also increased in *ct2* samples albeit by about only half (195.0 µS cm^–1^). A similar withered leaf was found with rice (Fig. 5). Nipponbare leaves became markedly brown, while DK22 leaves remained green. NaCl caused more leaf senescence in Nipponbare than DK22 (Fig. 5A). Photosynthesis activity was reduced during the early stage of the NaCl response and was quantified by chlorophyll degradation. NaCl (75mM) decreased chlorophyll by 47.1% in Nipponbare leaves, but had no significant effect in DK22 leaves (Fig. 5B). These data suggest that loss-of-function alleles of the Gα gene attenuate cellular senescence caused by high salinity.

### Loss of Gα does not alter ion composition in shoots

Sodium is taken up by roots and delivered from roots to shoots. Sodium transported to the shoots mostly remains in the tissue due to the lower level of transportation of sodium by phloem (Munns and Tester, 2008). To assess Gα function in the sodium uptake and delivery events, we quantitated sodium and potassium ions transported to shoots (Table 1). Sodium accumulated both in B73 and Gα-null *ct2* shoots to the same level (B73, 27.3mg g^–1^ DW; *ct2*, 29.9mg g^–1^ DW) under 100mM NaCl treatment, while potassium decreased in seedlings (B73, 25.3mg g^–1^ DW; *ct2*, 26.8mg g^–1^ DW). Similar sodium accumulation and potassium loss were observed in rice shoots with no significant difference between Nipponbare and Gα-null DK22 seedlings (Table 1). These results suggest that the Gα signalling pathway attenuated leaf senescence without altering sodium accumulation in shoots.

**Table 1.** Ion content in maize and rice shoots treated with NaCl^a^

		Sodium (mg g^–1^ DW)	Potassium (mg g^–1^ DW)	Sodium:Potassium ratio
B73	Control	0.13±0.03 (4)	54.5±5.0 (4)	n/a
	100mM NaCl	27.3±2.1 (5)	25.3±2.7 (5)	1.10±0.05 (5)
*ct2*	Control	0.10±0.01 (3) n.s.	66.9±4.3 (3) n.s.	n/a
	100mM NaCl	29.9±2.3 (3) n.s.	26.8±0.4 (3) n.s.	1.11±0.09 (3) n.s.
Nipponbare	Control	0.09±0.02 (5)	26.2±2.0 (5)	n/a
	75mM NaCl	1.31±0.16 (5)	17.9±3.2 (5)	0.08±0.02 (5)
DK22	Control	0.09±0.16 (5) n.s.	28.3±4.1 (5) n.s.	n/a
	75mM NaCl	1.15±0.28 (3) n.s.	23.7±1.2 (3) n.s.	0.05±0.01 (3) n.s.

^a^ Amounts of sodium and potassium ions in shoots were provided as milligrams per shoot mass (grams DW). Data are means ± SE. Numbers in parentheses represent the numbers of seedlings examined. n.s. indicates that the ion content was not significantly different between wild-type (B73 or Nipponbare) and Gα-null (*ct2* or DK22) groups at a *P* value of 0.05 (one-way ANOVA with the Tukey’s multiple comparison test). n/a means not analysed. Raw data are provided in Supplementary Figure S5.

### Gα signalling mediates sodium-specific toxicity

**Fig. 6.**
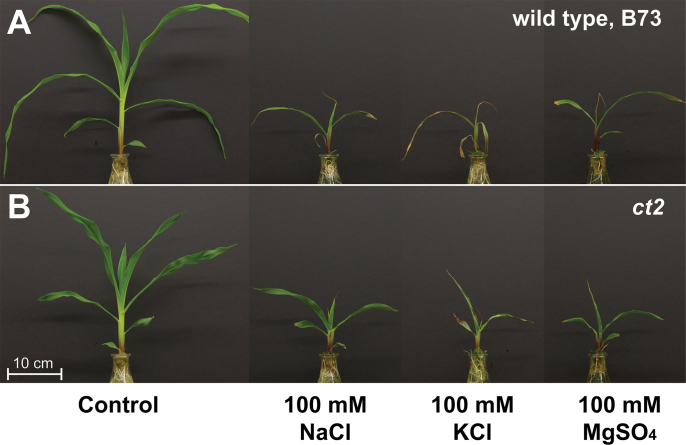
Effect of different salts on shoot growth. (A) Wild-type maize B73 or (B) Gα-null maize *ct2* seedlings were treated with or without 100mM NaCl, 100mM KCl, or 100mM MgSO_4_ in 1/4X MS liquid media for 10 d.

Shoot growth inhibition by NaCl is mainly due to the osmotic/ionic stress rather than sodium toxicity (Munns and Tester, 2008; Taleisnik *et al.*, 2009; Geng *et al.*, 2013), while the stress-induced leaf senescence is due to sodium toxicity. To dissect the Gα function between osmotic- and sodium-specific responses, we included 100mM KCl and 100mM MgSO_4_ (Fig. 6 and Table 2). KCl and MgSO_4_, as well as NaCl, inhibited seedling growth (Fig. 6) and shortened the third leaf sheath (Table 2) of B73 and *ct2* seedlings, revealing an osmotic effect on Gα-mediated control of cell division. On the other hand, the three salts had different effects on leaf senescence (Fig. 6 and Table 2). B73 leaves withered and became chlorotic with NaCl and KCl, while leaves treated with MgSO_4_ had brown edges and tips but otherwise stayed dark green. KCl and NaCl strongly increased electrolyte leakage; however, MgSO_4_ weakly increased the electrolyte leakage (Table 2). Taken together, NaCl inhibits seedling growth mainly through osmotic stress whereas cell senescence is through sodium toxicity. Both responses involve Gα.

**Table 2.** Effect of different salts on seedling growth and electrolyte leakage^a^

		Shoot FW (g)	Third leaf sheath (mm)	Third leaf blade (mm)	Conductivity (µS cm^–1^)
B73	Control	10.01±0.57 (13)	99.3±2.1 (13)	251.6±8.3 (13)	23.0±1.4 (26)
	NaCl	1.28±0.22 (12)	41.9±3.3 (12)	206.2±5.9 (12)	433.1±34.9 (24)
	KCl	1.33±0.05 (6)	52.3±1.4 (6)	210.8±4.5 (6)	827.8±77.0 (12)
	MgSO_4_	1.54±0.16 (6)	47.2±2.9 (6)	211.3±3.5 (6)	55.2±2.8 (12)
*ct2*	Control	9.30±0.70 (7) n.s.	68.0±3.2 (7) ††	172.0±10.9 (7) ††	29.8±2.8 (14) n.s.
	NaCl	3.33±0.72 (7) †	49.3±1.5 (7) n.s.	152.3±2.0 (7) ††	195.0±20.6 (14) ††
	KCl	1.94±0.17 (4) n.s.	41.8±3.1 (4) n.s.	144.8±6.2 (4) ††	688.3±36.7 (8) n.s.
	MgSO_4_	2.12±0.21 (4) n.s.	39.8±1.5 (4) n.s.	149.8±6.6 (4) ††	54.0±3.5 (8) n.s.

^a^ Wild-type (B73) or Gα-null (*ct2*) maize seedlings were treated with 100mM NaCl, 100mM KCl, or 100mM MgSO_4_. Data are means ± SE. Values of control and 100mM NaCl groups are as shown in Figs 2A, C, E, and 4B. Numbers in parentheses represent the numbers of seedlings or leaf disks measured. † or †† indicates significant differences between B73 and *ct2* groups at a *P* value <0.05 or 0.01 (one-way ANOVA with the Tukey’s multiple comparison test). n.s. means no significant difference at a *P* value of 0.05. Raw data are shown in Supplementary Figure S6.

## Discussion

**Fig. 7.**
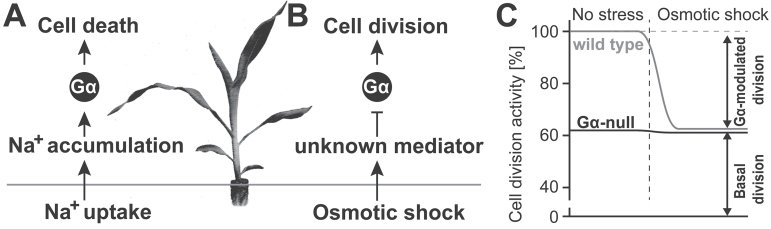
Proposed function of Gα on salt stress resistance. NaCl stress is divided into two functions: osmotic stress and sodium toxicity. (A) Sodium is taken up by roots and delivered to shoots. The Gα pathway does not control these events, because sodium accumulated to similar level in wild-type and Gα-null shoots. Sodium accumulation induces cellular senescence. We propose that the Gα pathway promotes cell death upon sodium accumulation. (B) High osmolality suppresses plant growth by inhibiting cell division and elongation. The process is perhaps mediated by unknown mediators, such as hormones. We propose that the mediator suppresses cell cycling through inhibition of Gα signalling. (C) Gα may function as a stress-responsive cell cycle modulator. In this model, there are two fractions of cell division activities; a Gα-independent basal cell division and a Gα-mediated cell division. The Gα-mediated fraction is flexibly modulated to adapt to the extracellular environmental change. For example, plants may inhibit the Gα-mediated cell division and reduce the shoot growth to adapt to shortage of water usage caused by high salinity.

We propose two distinct Gα functions in osmolality-induced growth inhibition and in ion-specific leaf senescence (Fig. 7A, B) and discuss G-protein functions across three distinct models: *Arabidopsis*, rice, and maize.

The Gα subunit is a stress responsive modulator of cell division. The Gα-null alleles in *Arabidopsis*, rice, and maize are dwarf; in the case of *Arabidopsis*, this phenotype is observed with etiolated seedlings (Fujisawa *et al.*, 1999; Ullah *et al.*, 2001; Bommert *et al.*, 2013). The Gα mutants reduced cell division in the leaf-length axis (Ullah *et al.*, 2001; Bommert et al., 2013; Izawa *et al.*, 2010; Urano *et al.*, 2013) but not in the leaf-width axis, suggesting their function in cell proliferation specifically in the shoot apical meristem and leaf primordia but not at the leaf plate meristem (Tsukaya, 2006). NaCl suppresses both cell division and cell expansion (Munns and Tester, 2008). Cellular length decreased with NaCl both in wild-type and Gα-null plants (Fig. 3B), while cell number was reduced only in the wild type to the basal level of a Gα-ablated plant (Fig. 3C). The Gα-independent fraction of cell division was not further inhibited by NaCl. In other words, the lack of a functional Gα gene phenocopied the inhibitory effect of NaCl on cell division, suggesting that Gα modulates a fraction of the cell division rate potential, a fraction which is also modulated by environmental factors such as high salinity (Fig. 7C). Inhibition of cell division by NaCl is due to its osmotic effect rather than sodium-specific toxicity (Table 2). The root, a sensory organ of osmolality, is spatially separated from the shoot apical meristem. High salinity increases or decreases some hormones and sugar metabolites (Zhu, 2002; Wang *et al.*, 2013; Colebrook *et al.*, 2014), although none of these has been proven to suppress cell division under high salinity. An inter-organ mediator that suppresses cell proliferation through a Gα-signalling pathway remains unknown.

High salinity causes leaf senescence by ion-specific toxicity rather than osmotic stress (Munns and Tester, 2008). Loss-of-function mutants of the Gα gene attenuated leaf senescence (Figs. 3 and 4) without altering sodium and potassium contents in leaves (Table 1), suggesting that Gα promotes cellular senescence after sodium reaches the toxic threshold. Leaf cells compartmentalize sodium in vacuoles to maintain a low cytoplasmic sodium concentration (Munns and Tester, 2008). Maize lacking the Gα subunit had less electrolyte leakage with NaCl than the wild type, whereas the effect of KCl or MgSO_4_ treatment was the same for both wild-type and Gα-mutant leaves (Table 2). This implies that Gα functions specifically in sodium compartmentalization or some other sodium-specific responses. *Arabidopsis* G subunits modulate programmed cell death promoted by biotic stresses (Llorente *et al.*, 2005; Trusov *et al.*, 2006) and abiotic stresses such as drought, salt, and ozone (Wang *et al.*, 2001; Booker *et al.*, 2004; Joo *et al.*, 2005; Chen and Brandizzi, 2012; Colaneri *et al.*, 2014;). *Arabidopsis* Gα mutants (*gpa1*), as observed here for rice and maize mutants, have attenuated chlorosis under salt stress, while the Gβ null mutation enhances chlorosis and death (Colaneri *et al.*, 2014). A role in multiple stress responses suggests that the G-protein network modulates programmed cell death more generally. We recently showed that sugar attenuates NaCl-triggered chlorosis in *Arabidopsis* (Colaneri *et al.*, 2014). High salinity increases soluble sugars in leaves (Misic *et al.*, 2012; Wang *et al.*, 2013). Because the *Arabidopsis* G-protein network mediates sugar responses (Chen *et al.*, 2003; Fu *et al.*, 2014; Urano *et al.*, 2012a), the Gα mechanism in osmotic and NaCl stress responses may be indirect through glucose sensing.

The *Arabidopsis rgs1* mutant, like the *gpa1* mutant, suppressed chlorosis under high salinity (Colaneri *et al.*, 2014), implying a genetic connection between the two proteins in a salt response pathway. Grasses lack a 7TM-RGS1 homologue of the *Arabidopsis* RGS1 gene, presumably altering how Gα activation is regulated, however, at least in the case of osmotic and ionic stress responses, the Gα subunit plays the same role in dicots and grasses.

In conclusion, the G-protein signalling network modulates cell division in meristems and regulates cell death in leaves in response to salt stress.

## Supplementary material

Supplementary data can be found at *JXB* online.

Supplementary Figure S1. Root length of maize seedlings grown with NaCl.

Supplementary Figure S2. Raw data plot for FW and leaf length of maize and rice seedlings grown with NaCl.

Supplementary Figure S3. Raw data plot for cell expansion and division in the maize sheath.

Supplementary Figure S4. Raw data plot for cellular senescence of the maize and rice leaves.

Supplementary Figure S5. Data plot for ion content in maize and rice seedlings treated with or without NaCl.

Supplementary Figure S6. Raw data plot for cellular senescence of the maize and rice leaves.

## Funding

This work was supported by grants from the NIGMS (R01GM065989) and NSF (MCB-0718202) to A.M.J. The Division of Chemical Sciences, Geosciences, and Biosciences, Office of Basic Energy Sciences of the US Department of Energy through the grant DE-FG02-05ER15671 to A.M.J. funded technical support in this study.

## Supplementary Material

Supplementary Data
