# Supplementary material for: Gα modulates salt-induced cellular senescence and cell division in rice and maize
Source: J Exp Bot. 2014 Sep 16;65(22):6553–61. doi: 10.1093/jxb/eru372 (PMC4246186; doi:10.1093/jxb/eru372)
Supplement: Supplementary Data [file supp_65_22_6553__index.html]

Gα modulates salt-induced cellular senescence and cell division in rice and maize — Gα modulates salt-induced cellular senescence and cell division in rice and maize — Supplementary Data 

# Gα modulates salt-induced cellular senescence and cell division in rice and maize

## Supplementary Data

Data files

**Files in this Data Supplement:**

- Supplementary Data - Supplementary Data
