# Supplementary material for: Gα modulates salt-induced cellular senescence and cell division in rice and maize
Source: J Exp Bot. 2014 Sep 16;65(22):6553–61. doi: 10.1093/jxb/eru372 (PMC4246186; doi:10.1093/jxb/eru372)
Supplement: Supplementary Data [file supp_eru372_jexbot129445_file001.pdf]

**Supplementary information for Urano, Colaneri and Jones**

Supplementary figure 1 illustrates the NaCl effect on root growth of maize. Supplementary figures 2-6 plot raw data that are explained in the main text and figures. Figures present raw values of each measurement, the means and the error bars representing 95% confidence interval. Wild type groups (Nipponbare or B73) or Gα-null groups (DK22 and *ct2*) are presented by orange or blue color.

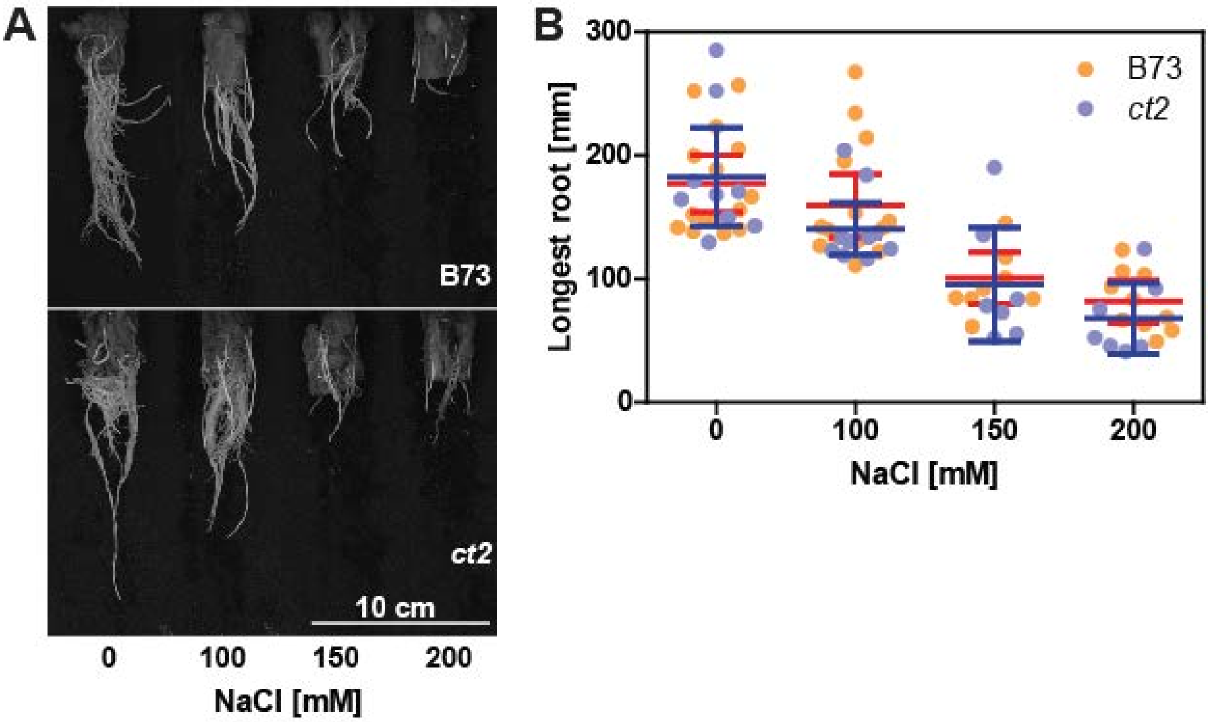


**Figure S1 Root length of maize seedlings grown with NaCl**

(**A**) Representative root pictures of wild type B73 and Gα null *ct2* seedlings grown with the indicated concentration of NaCl for 10 d. See figure 1 for pictures of shoots. (**B**) Longest root length of B73 or Gα-null *ct2* seedlings grown with NaCl for 10 d. Data are mean of seven or more root samples. Error bar represents 95% confidence interval. No significant difference was observed between B73 and *ct2* groups at the p value of 0.05 (One-way ANOVA with the Tukey's multiple comparison test).

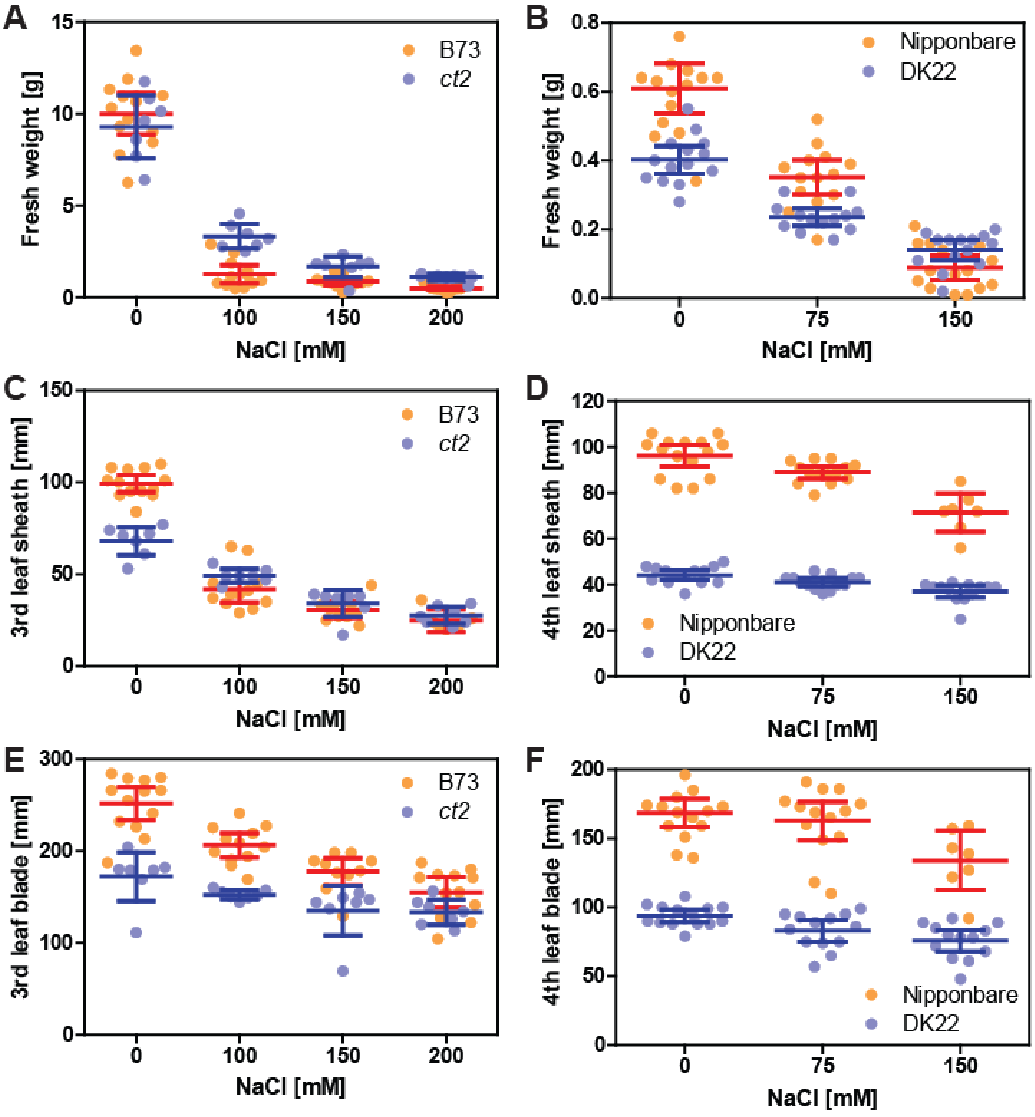


**Figure S2 Raw data plot for fresh weight and leaf length of maize and rice seedlings grown with NaCl**

(**A, B**) Fresh weight of maize or rice shoots after 10 d treatment with or without NaCl. (**C-F**) Length of the leaf sheath and blade of maize or rice seedlings grown with or without NaCl for 10 d. The third leaf of maize and the fourth leaf of rice were analyzed. Error bar represents 95% confidence interval. See figure 2 for statistical analyses.

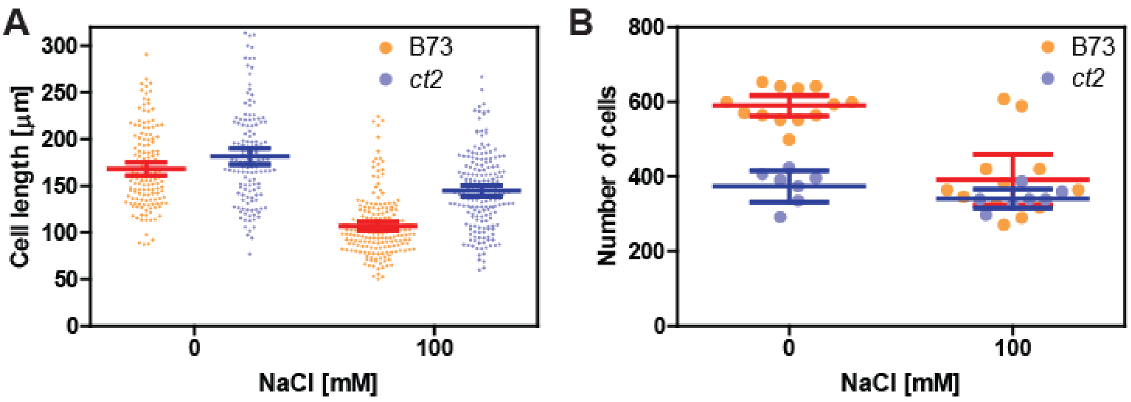


**Figure S3 Raw data plot for cell expansion and division in the maize sheath**

(**A**) Length of leaf and sheath epidermal cells after 10 d treatment with NaCl. Data are averaged over 130 cells from four or more images. Data are same as Fig. 3A (**B**) Number of cells forming the third leaf sheath along the longitudinal axis. The number was estimated by dividing each sheath length by the averaged cellular length. Error bar represents 95% confidence interval. See figure 3 for statistical analyses.

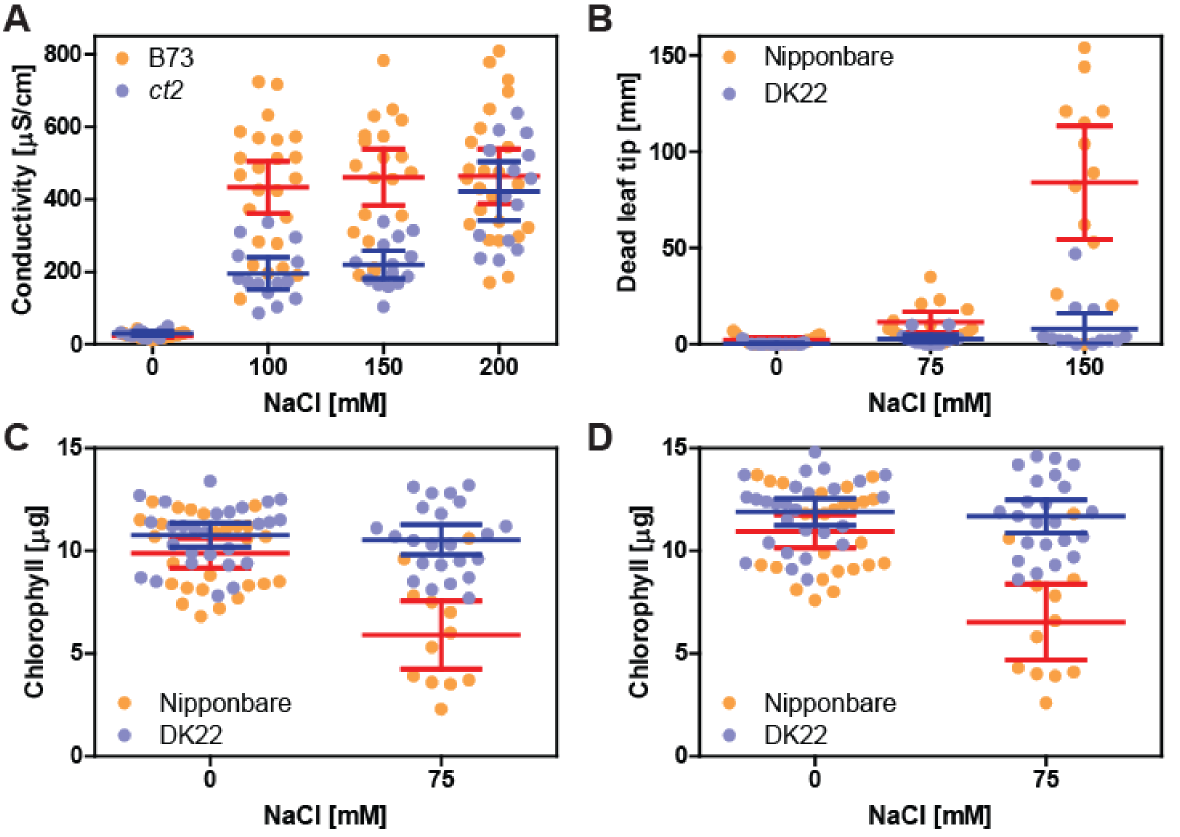


**Figure S4 Raw data plot for cellular senescence of the maize and rice leaves**

(A) Electrolyte leakage from the wild type or Gα-null maize leaf disks were measured. (B) Length of withered leaf tips of wild type or Gα-null rice grown with NaCl for 10 d. The second leaf blades were analyzed. (C, D) Total chlorophyll amount per two rice leaf disks (ϕ5 mm) was estimated with Lichtenthaler's equation (C) or Arnon's equation (D). Error bar represents 95% confidence interval. See figure 4 and 5 for statistical analyses.

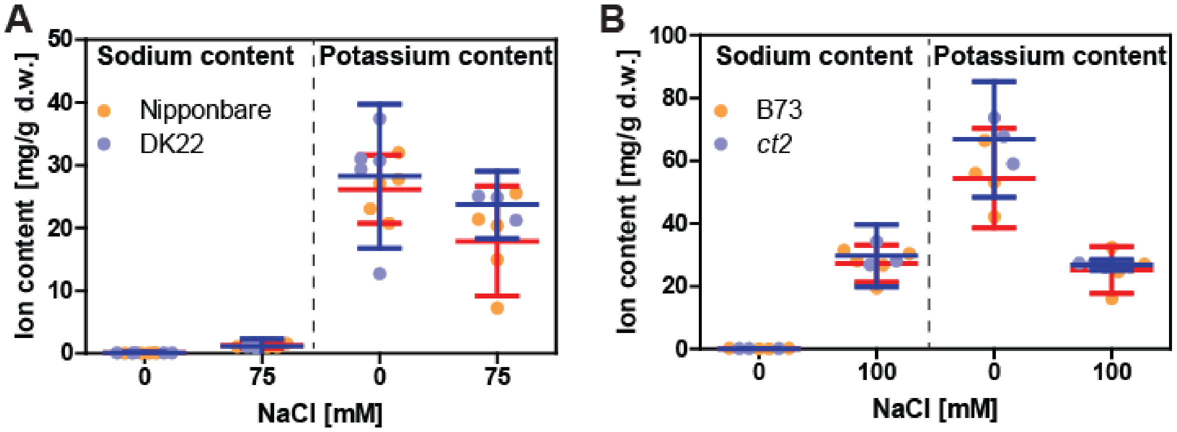


**Figure S5 Data plot for ion content in maize and rice seedlings treated with or without NaCl.**

Amounts of sodium and potassium ions in shoots were shown as milligram per shoot mass (gram of dry weight) for rice (**A**) and maize (**B**). Data are shown with 95% confidence interval. See table 1 for statistical analyses.

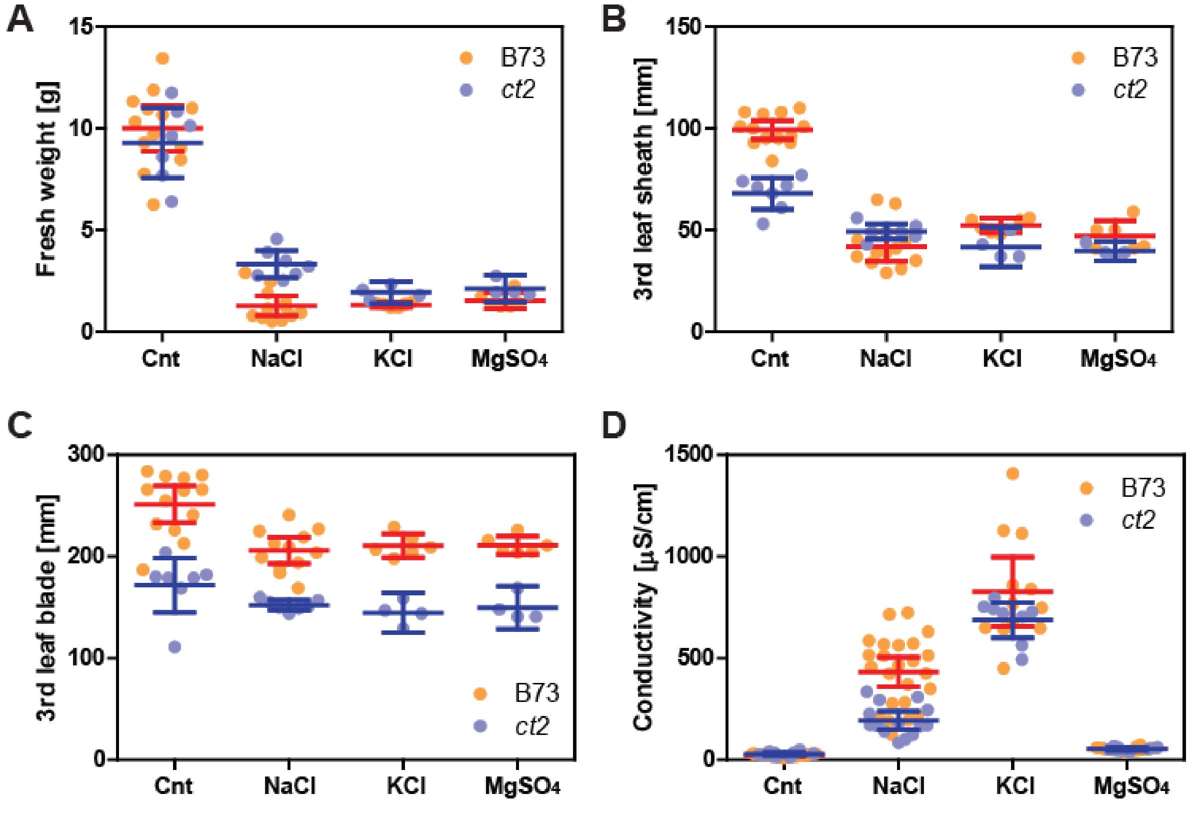


**Figure S6 Raw data plot for cellular senescence of the maize and rice leaves**

(**A**) Electrolyte leakage from the wild type or Gα-null maize leaf disks were measured. (**B**) Length of withered leaf tips of wild type or Gα-null rice grown with NaCl for 10 d. The second leaf blades were analyzed. (**C, D**) Total chlorophyll amount per two rice leaf disks (ϕ5 mm) was estimated with Lichtenthaler's equation (**C**) or Arnon's equation (**D**). Error bar represents 95% confidence interval. See figure 4 and 5 for statistical analyses.
